# A novel vasculogenic mimicry-related nomogram predicts prognosis in hepatocellular carcinoma

**DOI:** 10.3389/fgene.2025.1431624

**Published:** 2025-07-07

**Authors:** Yun Zhong, Fadian Ding, Han Zhang, Denghan Zhang, Xiang Zhang, Shangeng Weng

**Affiliations:** ^1^ Department of Hepatobiliary and Pancreatic Surgery, The First Affiliated Hospital, Fujian Medical University, Fuzhou, China; ^2^ Fujian Abdominal Surgery Research Institute, The First Affiliated Hospital, Fujian Medical University, Fuzhou, China; ^3^ Department of Hepatobiliary and Pancreatic Surgery, National Regional Medical Center, Binhai Campus of the First Affiliated Hospital, Fujian Medical University, Fuzhou, China; ^4^ Fujian Provincial Key Laboratory of Precision Medicine for Cancer, The First Affiliated Hospital, Fujian Medical University, Fuzhou, China

**Keywords:** hepatocellular carcinoma, vasculogenic mimicry, immune infiltration, immune checkpoints, prognosis prediction

## Abstract

**Objective:**

Hepatocellular carcinoma (HCC) is the most common type of liver cancer and has a poor prognosis. Vasculogenic mimicry (VM) is an angiogenic process associated with the growth and spread of malignant tumors. In this study, we aim to create a VM-related, gene-based prediction model to evaluate the prognosis and immune infiltration in HCC patients.

**Materials and methods:**

A total of 364 patients from the TCGA database and 242 patients from the GEO database with complete clinical information and transcriptome sequencing data were enrolled in this study. LASSO Cox regression analysis was performed to identify VM-related hub genes. Biological process (BP), Kyoto Encyclopedia of Genes and Genomes (KEGG) enrichment analysis, and gene set enrichment analysis (GSEA) were applied to analyze the biological function of the hub genes. The predictive significance of the related gene signature was confirmed in the GSE14520 cohort. RT-PCR and CD31/E-cadherin immunofluorescence staining were applied to elucidate that the VM score can reflect the degree of vasculogenic mimicry within tumors.

**Results:**

This study found that VM-related genes were enriched in the proteoglycans in the cancer pathway and the VEGF signaling pathway. A predictive signature based on five genes (*MAPK3*, *MAPK1*, *VEGFA*, *NOTCH1*, and *TGFB1*) was identified as an independent risk factor for HCC patient prognosis. GSEA revealed that genes that positively correlated with the signature were enriched in the “NOTCH signaling pathway,” which is activated during angiogenesis. Additionally, CIBERSORTx analysis showed that higher expression of the VM score was associated with immune infiltration of naïve CD4^+^ T cells in HCC. Pearson correlation analysis revealed a positive link between an increased VM score and inhibitory immunological checkpoints (HVEM and PD-1). Furthermore, *in vivo* experiments have confirmed that the VM score can effectively reflect the degree of vasculogenic mimicry in hepatocellular carcinoma tissue. The nomogram that utilized the VM score and TNM stage to predict the survival probability of individual HCC patients was satisfactory.

**Conclusion:**

The VM score and nomogram constructed to predict the survival probability of HCC patients achieved satisfactory outcomes in this study. The relationship between the biological function of the VM score and immune infiltration could potentially serve as a target for tumor therapy in liver cancer.

## 1 Introduction

Vasculogenic mimicry (VM) has emerged as a significant factor influencing the prognosis of hepatocellular carcinoma (HCC). In this study, we have innovatively elucidated and validated the correlation between the VM score derived from VM-related genes and the prognosis of HCC patients, alongside tumor immune infiltration. Through *in vitro* experiments, we also verified that the VM score can reflect the degree of vasculogenic mimicry within tumors. The prognostic signature can provide a basis for further study of vasculogenic mimicry in HCC and guidance for individualized treatment of HCC. Hepatocellular carcinoma (HCC), a primary tumor of the liver, ranks as the fifth most common cause of cancer-related deaths worldwide (Chidambaranathan-Reghupaty et al., 2021). It is an aggressive tumor with rapid growth, poor prognosis, and high mortality (Llovet et al., 2021). Surgical resection and liver transplantation are the primary treatments for patients with HCC (Ducreux et al., 2023). Unfortunately, the 5-year survival rate remains very low, at less than 20%, making HCC the second leading cause of cancer-related death after pancreatic cancer in the United States (Siegel et al., 2018). Therefore, novel therapeutic targets and biomarkers must be rapidly investigated to enhance patients’ prognoses.

To develop its own supply of nutrients and oxygen, a tumor needs to form new blood vessels (Lugano et al., 2020). Recent evidence suggests that endothelium-dependent tumor angiogenesis is the most common pathway of angiogenesis (Liu et al., 2023). Some anti-angiogenic targeted drugs depend on blocking this pathway to achieve positive clinical outcomes (Teleanu et al., 2019). However, the clinical efficiency of targeted therapy is still unsatisfactory. Existing research on angiogenesis suggests a new process called vasculogenic mimicry (VM) in many malignant tumors (Luo et al., 2020). VM is an epithelial-independent tumor angiogenesis pathway that is present in highly malignant tumors, including HCC (Wei et al., 2021). It is characterized by the formation of new vessels that contain endothelial tumor cells and matrix components (Kirschmann et al., 2012). Several studies have suggested an association between VM and targeted drug resistance in malignant tumors (Hori et al., 2019; Ge and Luo, 2018). An increasing number of studies are attempting to reveal the mechanism of VM. In addition, previous research has established that various VM-related genes, such as *VEGFA* and *LOXL2*, are involved in the VM process (Cao et al., 2013). Wang, H. F. reported that hypoxia can promote the VM process by regulating the expression of VM-related molecules, such as TWIST, LOX, and MMP2 (Wang et al., 2019). However, there have been no reports on these VM-related genes being involved in liver cancer.

In this study, we retrieved genes relevant to vasculogenic mimicry from previous research and explored their relationship between those genes and the prognosis in LIHC samples from the TCGA database. Hub genes associated with the VM process were selected through univariate and LASSO Cox regression analyses. Based on these, we established the VM score. Then, we examined the relationship between immune checkpoints and the VM score. Finally, a nomogram based on VM score and clinical characteristics was modeled to predict the prognosis of HCC patients. This study provides a novel biomarker for prognosis prediction and potential therapeutic targets in HCC.

## 2 Materials and methods

The RNA-seq data and clinical data of 377 LIHC patients were downloaded from the TCGA website (https://tcga-data.nci.nih.gov/tcga/). The GSE14520 dataset, containing gene expression data from 247 HCC patients, was downloaded from the GEO website (http://www.ncbi.nlm.nih.gov/geo/). Incomplete survival data and normal tissues were excluded, and 364 patients were randomly divided into a training group and a test group in a 7:3 ratio. A total of 254 patients from the TCGA database were enrolled in the training group, and 110 patients from the TCGA database were enrolled in the test group. Five patients with incomplete information were excluded, and 242 patients from the GEO database were included in the validation group.

### 2.1 Functional enrichment analysis of genes related to vasculogenic mimicry

Twenty-four VM-related genes were collected from the existing reviews, primarily those obtained from Yang et al. (2023) and Wang et al. (2022). The R package “pheatmap” was used to draw heatmaps. A protein–protein interaction (PPI) network was built by uploading the VM-related genes to the STRING database (https://string-db.org/). Gene Ontology (GO) and Kyoto Encyclopedia of Genes and Genomes (KEGG) enrichment analyses were performed using the DAVID Functional Annotation Bioinformatics Microarray Analysis (https://david.ncifcrf.gov/). The GO analysis includes three parts: biological process (BP), molecular function (MF), and cellular component (CC).

### 2.2 Construction and validation of the VM score in HCC

Based on the training group, VM-hub genes were screened using univariate Cox regression analysis through the “survival” R package. Additionally, LASSO Cox regression analysis was performed using the “glmnet” R package. The optimal λ-value is shown in Supplementary Figure S1. Consequently, five genes and their coefficients (*MAPK1*: 0.0133846666973586; *MAPK3*: 0.0407970850574295; *VEGFA*: 0.0170936015827322; *NOTCH1*: 0.0285005633980989; and *TGFB1*: 0.00206739390155587) were used to calculate the VM score. The VM score of each patient was calculated using the following formula:

VM score = *MAPK1* expression × 0.0133846666973586 + *MAPK3* expression × 0.0407970850574295 + *VEGFA* expression × 0.0170936015827322 + *NOTCH1* expression × 0.0285005633980989 + *TGFB1* expression × 0.00206739390155587.

We divided the patients into high- and low-risk groups using the best cutoff value determined through the “survminer” R package. The R packages “pheatmap,” “glmnet,” and “ggplot2” were used to show differences in clinical information between the high- and low-risk groups. Kaplan–Meier (KM) survival curves were plotted using the “survival” R package.

### 2.3 External validation of the VM score

The VM score of each patient included in the validation group was calculated using the same formula. The optimal cutoff value was also identified using the “survminer” R package. Based on this threshold, patients were divided into low- and high-risk groups. Differences in clinical information between the two groups were represented using the R packages “pheatmap,” “glmnet,” and “ggplot2.” The gene set enrichment analysis (GSEA) based on the “clusterProfiler” and “patchwork” R packages was applied to evaluate the VM score-related biological functions and involved pathways. GEPIA was used to visualize the VM score-related genes identified through GO and KEGG analyses.

### 2.4 Evaluation of immune cell infiltration and VM-related immune checkpoints

CIBERSORTx (https://cibersortx.stanford.edu/) was used to explore the potential correlation between the signature and immune cell infiltration in the TCGA and GEO databases (Newman et al., 2015). The correlation between the VM score and immune cells was represented using the “ggpubr” and “ggExtra” R packages. In addition, we explored the relationship between the VM score and immune checkpoints in the TCGA and GEO databases. The “oncoPredict” R package was used to analyze the differences in drug sensitivity between high- and low-VM score groups.

### 2.5 Nomogram construction

Cox multivariate analysis was performed to determine the independent prognostic clinical factors. The “rms” R package was used to build a predictive nomogram in the training group. The 1-, 3-, and 5-year survival rates of HCC patients were calculated based on the total points. In addition, the receiver operating characteristic (ROC) curve of the nomogram was plotted using the “timeROC” R package. Calibration curves assessed the accuracy of the prediction model. The prediction accuracy was validated in the validation group.

### 2.6 Reverse transcription quantitative polymerase chain reaction

Total RNA was extracted from the designated source, and complementary DNA (cDNA) was synthesized using a reverse transcription kit [HiScript® III RT SuperMix for qPCR (+gDNA wiper) (Vazyme, China)]. Specific primers targeting the desired genes, both target and reference genes, were meticulously designed and subsequently validated. Quantitative polymerase chain reaction (qPCR) assays were conducted in triplicate using a SYBR Green master mix. Modifications in mRNA levels were assessed using the 2^−ΔΔCT^ methodology, with β-actin as the internal reference for normalization. Statistical analysis was conducted to determine significance.

### 2.7 Animal experiments

Ethical approval was obtained for this retrospective study (IACUC FJMU 2023-0333). An *in situ* liver cancer model was induced in male C57BL/6J mice, aged 4 weeks, comprising a total of 20 subjects. Male C57BL/6 mice were purchased from Fuzhou zolgene biotechnology. To establish the *in situ* liver cancer model, 1 × 10^6^ Hepa 1-6 cells were directly injected into the liver tissue of each mouse. Tissue specimens were collected 3 weeks following injection for the assessment of tumor angiogenesis. Anesthesia was administered in accordance with established protocols.

### 2.8 Immunofluorescence staining

Immunofluorescence staining was used to assess protein expression levels in formalin-fixed, paraffin-embedded tissue sections. The sections were blocked and then incubated with primary antibodies against CD31 (CD31 Polyclonal antibody, # 28083-1-AP) and E-cadherin (E-cadherin Polyclonal antibody, # 20874-1-AP) overnight at 4°C and then washed. After washing, fluorescent secondary antibodies were applied. Subsequent steps included incubation with a secondary antibody and DAB substrate. Afterward, the sections were mounted and imaged using a fluorescence microscope. The acquisition of images and their analysis for semi-quantitative purposes were conducted using ImageJ software. Statistical analyses were rigorously carried out to compare protein expression levels among different experimental groups, ensuring compliance with ethical guidelines.

### 2.9 Statistical analysis

Statistical analyses were performed using R software (version 4.3.1; https://www.r-project.org/). The prognostic value was evaluated through Kaplan–Meier and Cox multivariate analyses. Pearson correlation analysis was used in our study. P < 0.05 was considered significant.

## 3 Results

### 3.1 Expression level of VM-related genes and enrichment analysis

**FIGURE 1:**
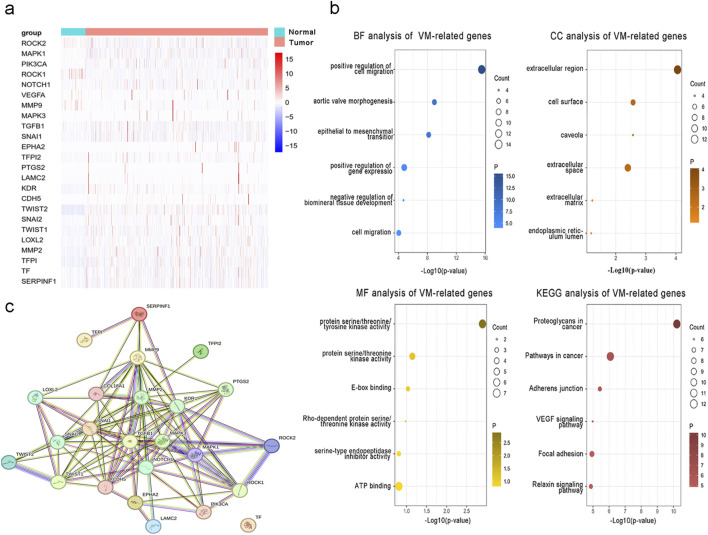
Expression and function of 24 VM-related genes in hepatocellular carcinoma. **(a)** Expression of VM-related genes between tumor and normal tissues. **(b)** GO and KEGG enrichment analyses of VM-related genes. **(c)** PPI network of VM-related genes.

**FIGURE 2:**
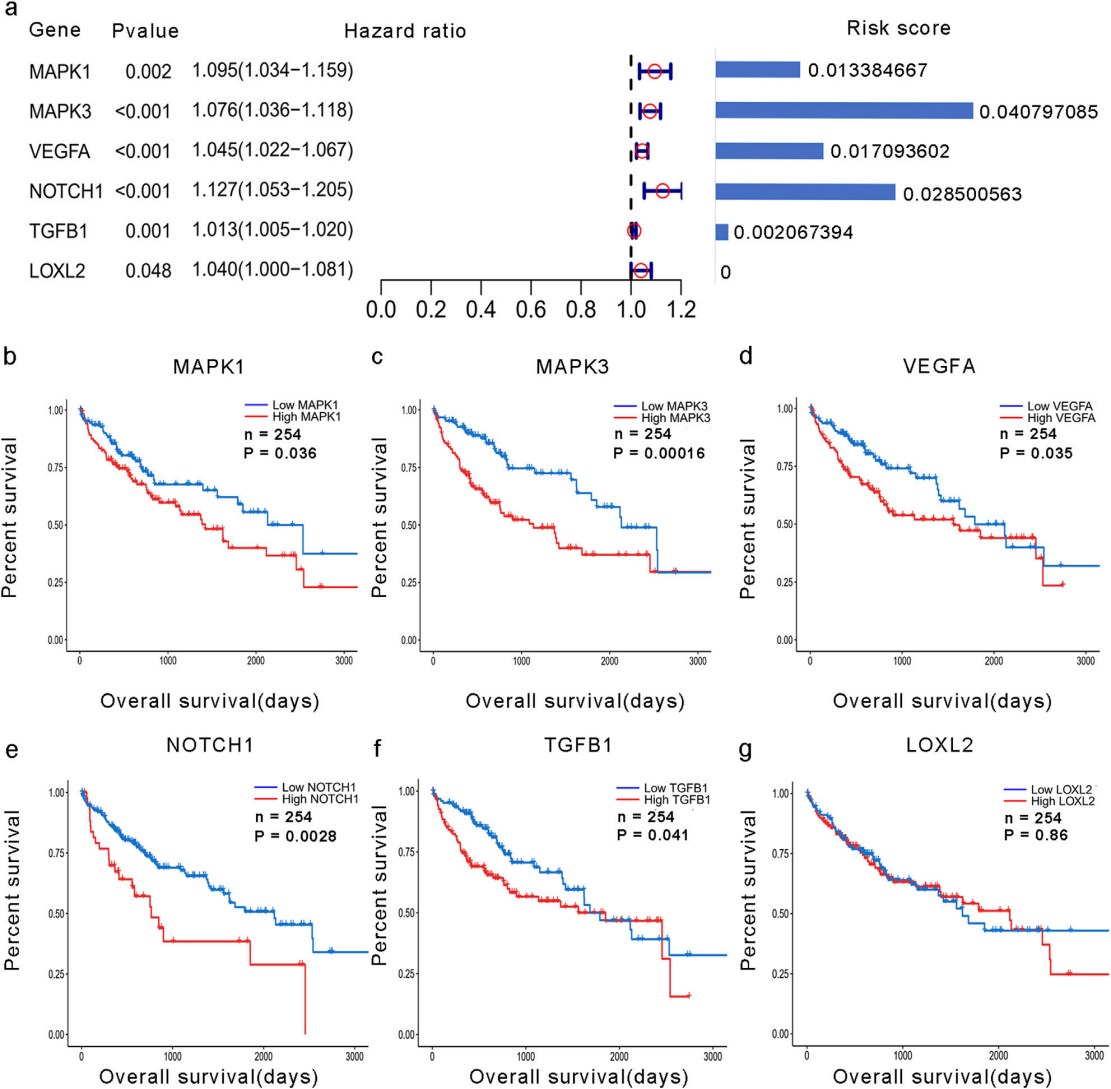
Development of the VM score. **(a)** Forest plot of the univariate analysis of VM-related genes and their coefficients. **(b–g)** KM analysis of overall survival in groups with high expression of hub VM-related genes and low expression of hub VM-related genes in the TCGA dataset.

To examine the function and expression of VM-related genes in liver cancer, a comparative analysis was performed on gene expression in normal tissue and liver cancer tissue sections. The heatmap shows elevated expression of VM-related genes in HCC (Figure 1a). The PPI network of VM-related genes is shown in Figure 1c. Subsequently, we carried out GO and KEGG enrichment analyses to investigate the potential functions and pathways for 24 VM-related genes. The KEGG analysis results showed that the top six involved pathways were “proteoglycans in cancer,” “pathways in cancer,” “adherens junction,” “VEGF signaling pathway,” “focal adhesion,” and “relaxin signaling pathway.” The top six GO analysis results were as follows: BP: positive regulation of cell migration, aortic valve morphogenesis, epithelial-to-mesenchymal transition, positive regulation of gene expression, negative regulation of biomineral tissue development, and cell migration; MF: protein serine/threonine/tyrosine kinase activity, protein serine/threonine kinase activity, E-box binding, Rho-dependent protein serine/threonine kinase activity, serine-type endopeptidase inhibitor activity, and ATP binding; and CC: extracellular region, cell surface, caveola, extracellular space, extracellular matrix, and endoplasmic reticulum lumen (Figure 1b). We found that MAPK1, MAPK3, VEGFA, NOTCH1, TGFB1, and LOXL2 were highly expressed in patients with poor prognoses. In addition, the Kaplan–Meier curves based on the TCGA database showed that the expression of these genes was negatively correlated with prognosis in liver cancer (Figures 2b–f).

### 3.2 Development of a prognostic signature for hepatocellular carcinoma

**FIGURE 3:**
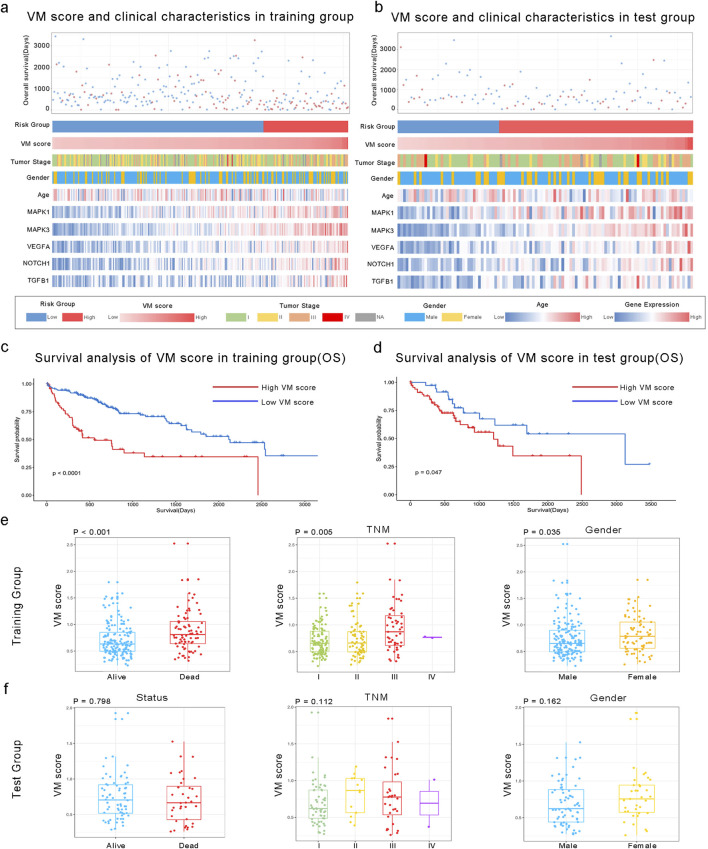
Association between the VM score and clinical characteristics of HCC. **(a, b)** Landscape of VM score-related clinical characteristics of HCC in training and test groups of the TCGA dataset. **(c, d)** KM analysis of overall survival in high and low VM score groups based on the TCGA dataset. **(e, f)** Differences in the VM score based on clinical characteristics, including status, TNM stage, and gender. The VM score was significantly increased in HCC patients with poor prognoses, those with a higher TNM stage, and in female patients in the training group but not in the test group.

A total of 24 VM-related genes were included in the univariate Cox regression analysis, and six prognostic genes were screened. Subsequently, a prognostic signature called the VM score was constructed through LASSO Cox analysis. The results showed that MAPK3, MAPK1, VEGFA, NOTCH1, and TGFB1 were risk factors in LIHC (Figure 2a). Finally, five hub genes and their risk scores were used to calculate the points. The optimal cutoff point identified using the “survminer” R package was applied to classify HCC patients into low- and high-risk groups. Patients in the different groups exhibited different clinicopathological characteristics. In the training and test groups, TNM stage, gender, and age showed asymmetric distributions with the increasing VM score (Figures 3a,b). KM curves for the training and test groups are shown in Figures 3c,d, indicating that a higher VM score was correlated with a poor prognosis. The VM score showed superior predictive performance for overall survival in training and test groups. The relationship between the VM score and clinical characteristics was further analyzed. As shown in Figures 3e,f, the VM score significantly increased in patients with poor prognoses, low-grade tumors, and female patients in the training group. However, there was no significant difference between the above subgroups in the test group.

### 3.3 External validation of the VM score in the GEO database

**FIGURE 4:**
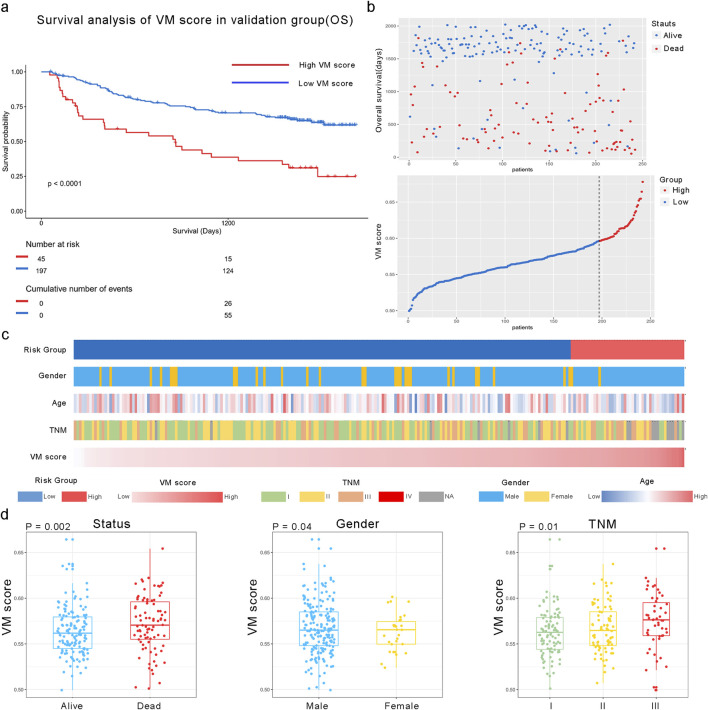
Relationship between the VM score and clinical characteristics in the GEO dataset. **(a)** KM analysis of overall survival in high and low VM score groups in GSE14520. **(b)** All patients were divided into high and low VM score groups based on the best cutoff value in the GEO dataset. **(c)** Heatmap of VM score-related clinical characteristics in the validation group. **(d)** Different distributions of the VM score based on different clinical characteristics; the VM score was significantly increased in HCC patients with poor prognosis, those with higher TNM stage, and female patients in the validation group.

**FIGURE 5:**
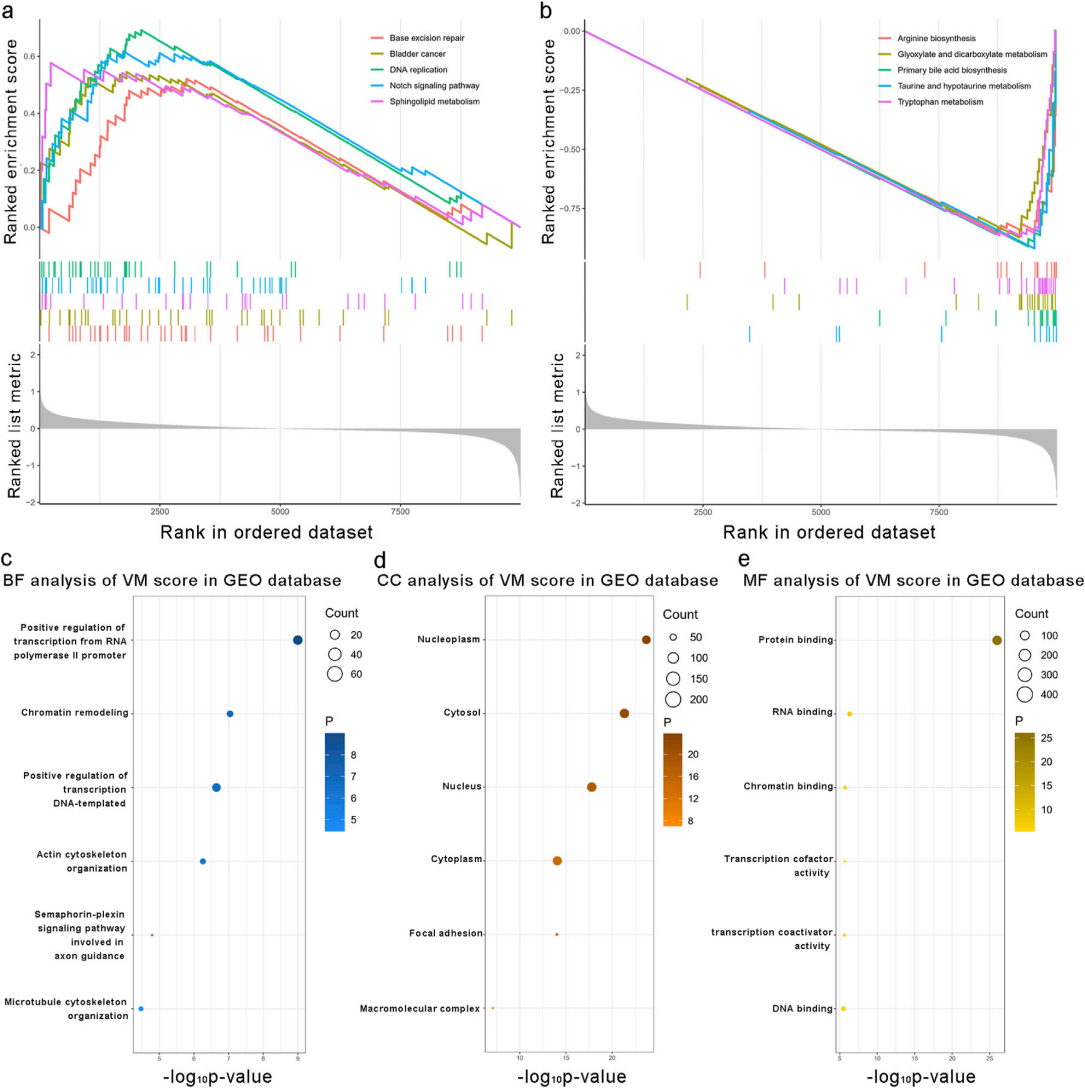
Biological functions associated with the VM score. **(a, b)** Gene set enrichment analysis results of the enrichment pathways associated with the VM score in the validation group. **(c–e)** Mostly related biological processes, cellular components, and molecular functions to the VM score in the validation group. Kyoto Encyclopedia of Genes and Genomes enrichment analysis of the VM score in the validation group.

We applied the formula to HCC patients in GSE14520 to verify the predictive accuracy of the VM score. As shown in Figure 4, patients in the high-risk group had worse overall survival (OS) than those in the low-risk group (P < 0.0001). Additionally, the clinical characteristics, such as TNM stage, gender, and age, showed an asymmetric distribution similar to that of the TCGA database. The VM score significantly increased in patients with a poor prognosis and higher-grade TNM stage in the GEO database. Furthermore, the potential pathway involved in the VM score was validated through GSEA analysis, as shown in Figures 5a,b. We found that genes positively correlated with the VM score were enriched in the “NOTCH signaling pathway,” which is activated during angiogenesis. The GO analyses were performed to investigate biological functions. The results of the analysis are shown in Figures 5c–e; the VM score was closely related to “positive regulation of transcription from RNA polymerase II promoter” in biological processes in the validation group. The most prevalent molecular function of the VM score was “protein binding.”

### 3.4 The VM score and immune cell infiltration

**FIGURE 6:**
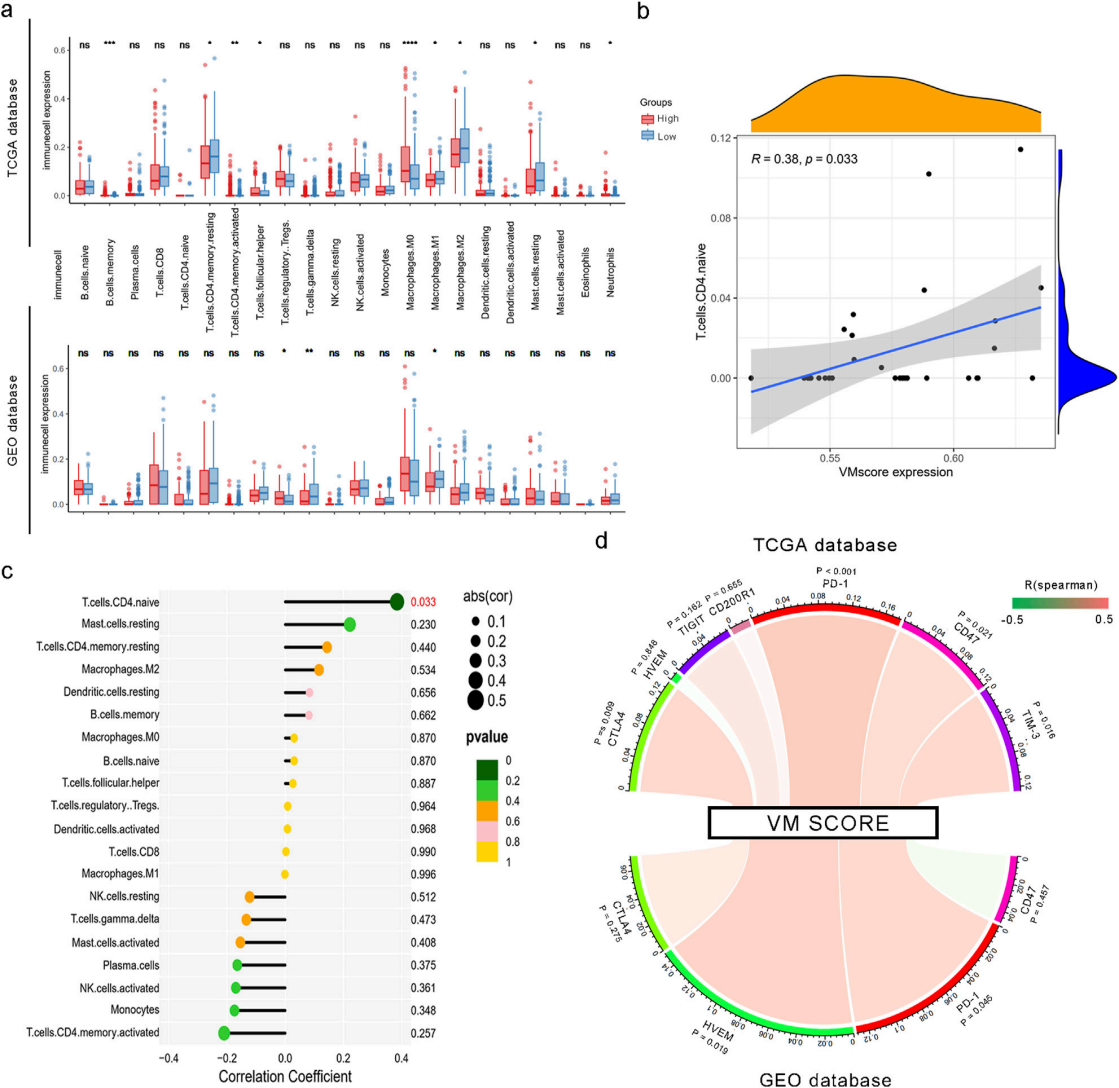
Correlation between the VM score and immune cell infiltration and checkpoints. **(a)** Infiltration of 22 types of immune cells in high and low VM score groups based on TCGA and GEO datasets. *, P < 0.05; **, P < 0.01; and ****, P < 0.0001. **(b)** Correlation of the VM score with the expression level of naïve CD4^+^ T cells. **(c)** Correlation of the VM score with immune cell infiltration based on CIBERSORTx. **(d)** Spearman’s rank correlation between the VM score and inhibitory immune checkpoints. The color of the band represents the R-value.

The correlation between the VM score and the immune landscape was tested using CIBERSORTx, and the result showed that the distribution and number of immune cells within tumors were significantly different among patients. As shown in Figure 6a, the CIBERSORTx results showed that CD4 T memory cells, CD8 cells, and macrophages were enriched in tumor tissue. We then compared immune infiltration in the high- and low-VM score groups and found that CD4 memory cells, M1 macrophages, and M2 macrophages were positively correlated with the VM score. M0 macrophages were negatively correlated with the VM score in the TCGA database. In the GEO database, higher degrees of infiltration were observed in M0 macrophages and regulatory T cells (Tregs), while gamma delta T cells showed the opposite. Furthermore, we analyzed 31 patients with significant differences in immune infiltration in the GEO database and found that CD4 naïve T cells were positively correlated with the signature (R = 0.38; P = 0.033) Figures 6b,c. Subsequently, we discovered a positive relationship between the VM score and seven known inhibitory immune checkpoints, namely, CTLA4, HVEM, TIGIT, CD200R1, PD-1, CD47, and TIM-3, in the training group (Figure 6d). Limited by the insufficient number of sequenced genes, only four checkpoints were investigated in the GEO database. PD-1 exhibited the same positive relationship with the VM score (Figure 6d). Additionally, we conducted drug sensitivity testing for VEGF inhibitors in high- and low-VM score groups. The results showed that the IC_50_ values of sorafenib and cediranib were lower in the high VM score group than in the low VM score group in both the TCGA test set and the GEO validation set, suggesting a better practical value of our model (Supplementary Figure S2). Reasonably, the results indicated that the prognostic signature could be a good indicator for assessing immune infiltration and a new biomarker for guiding immunotherapy in HCC patients.

### 3.5 Construction and validation of the risk prediction model

**FIGURE 7:**
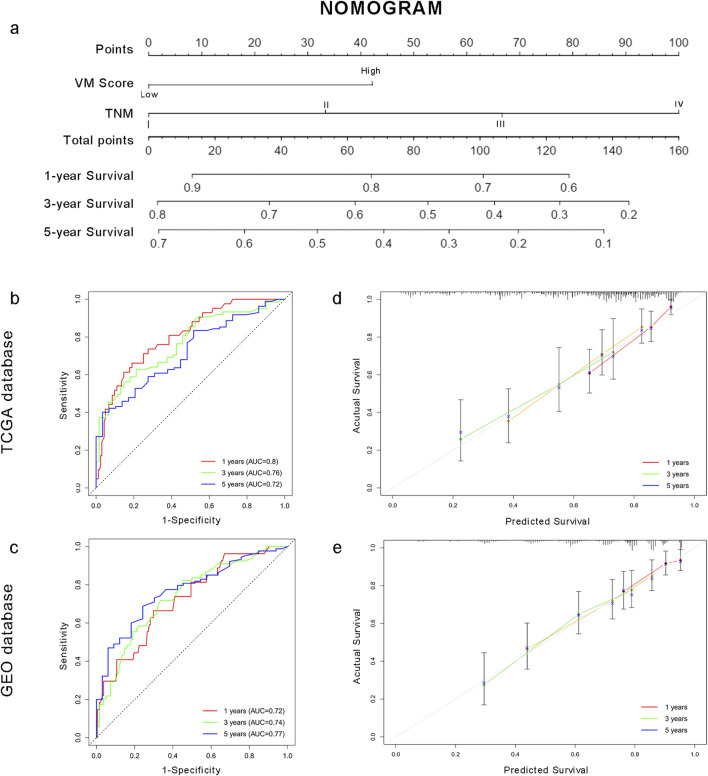
Individualized prediction model based on the VM score for HCC patients. **(a)** Nomogram for HCC patients. **(b–c)** Time-dependent ROC curves of the nomogram in the TCGA and GEO datasets. **(d–e)** Calibration curves of the nomogram. The plots show the comparison between predicted and actual OS for 1-, 3-, and 5-year survival probabilities in TCGA and GEO datasets.

To facilitate the clinical application of the VM score in HCC patients, we developed a prediction nomogram by combining the VM score and clinical characteristics (Figure 7a). Cox multivariate analysis indicated that the VM score and TNM stage are independent prognostic factors for overall survival (Supplementary Table S1). The individualized nomogram for predicting overall survival comprised the VM score and tumor stage. As shown in Figures 7b,c, the survival probabilities at 1, 3, and 5 years could be calculated using the prediction model. The 1-, 3-, and 5-year AUC values of the nomogram were 0.80, 0.76, and 0.72 in the training group and 0.72, 0.74, and 0.77 in the test group, respectively. The C-index of the nomogram was 0.661 in the TCGA dataset and 0.688 in the GEO dataset. Calibration curves showed that the predicted probabilities of the nomogram closely aligned with the actual survival probability estimates in both the training and test groups (Figures 7d,e). These results revealed that the nomogram could accurately predict the survival probability of HCC patients.

### 3.6 Validation of the relationship between the VM score and degree of vasculogenic mimicry *in vitro*

**FIGURE 8:**
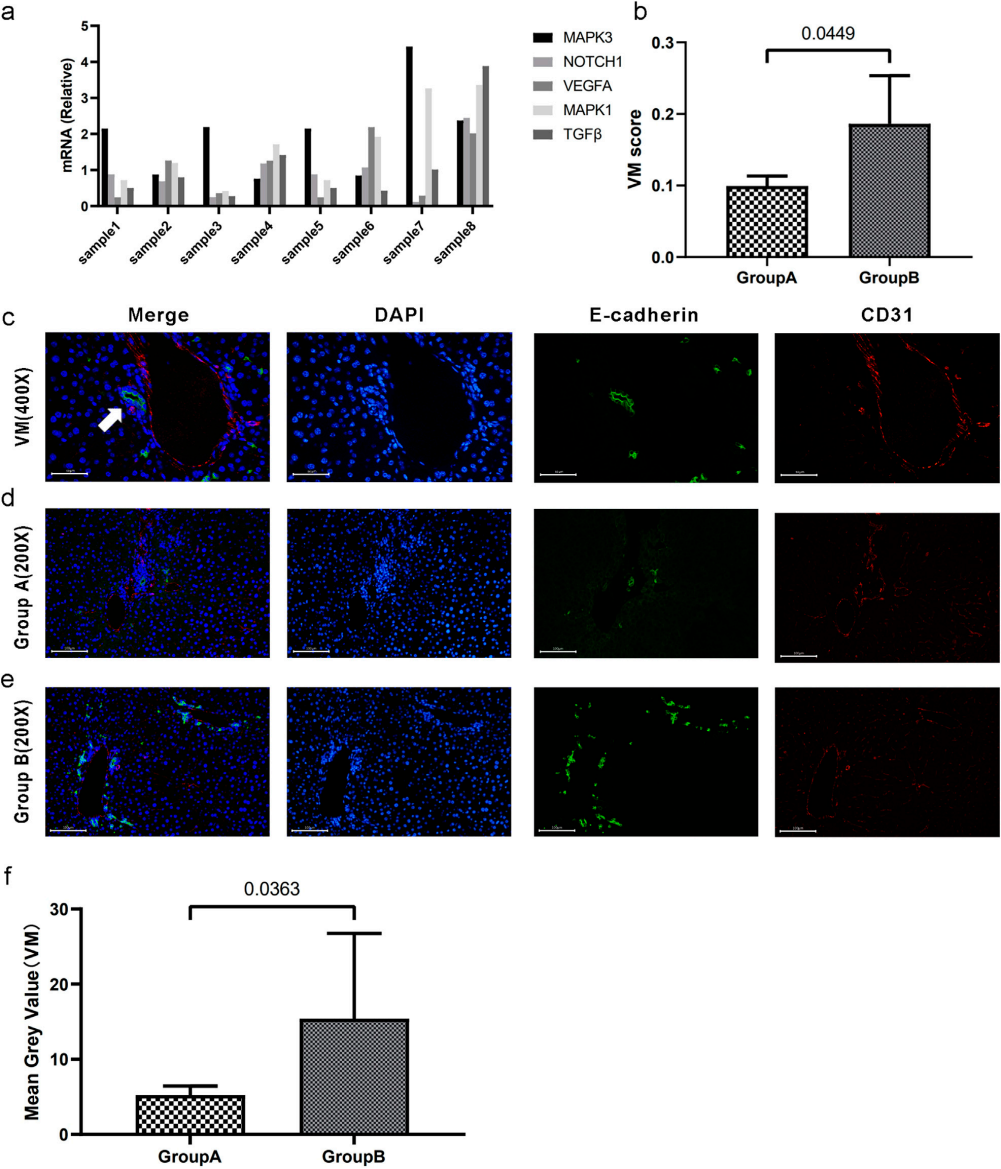
Validation of the relationship between the VM score and the degree of vasculogenic mimicry. **(a)** mRNA levels (relative quantification) of VM-related genes across eight samples. **(b)** Segregation into groups A and B based on the median VM score. There exists a statistically significant difference in VM scores between the two groups. **(c)** CD31/E-cadherin double staining for vasculogenic mimicry. The white arrow indicates tubular structures resembling VM. The lumens are surrounded by tumor tissue without endothelial cells. **(d–e)** CD31/E-cadherin double immunofluorescence staining of VM in tumor tissues in groups A and B. **(f)**. The statistical difference was confirmed using the Mann-Whitney U test. Data are expressed as means.

To elucidate the correlation between the VM score and the degree of tumor vasculogenic mimicry, we established orthotopic liver cancer models by directly injecting Hepa 1-6 cells into 20 C57BL/6 mice. The mRNA levels of VM-related genes in the 20 mice were measured using qPCR, and the corresponding VM scores were calculated (Figure 8a). The VM scores of these samples were arranged in descending order, and the top four samples were assigned to Group A, whereas the bottom four samples were assigned to Group B. The mRNA levels of the eight samples are shown in Figure 8. Figure 8b shows a statistically significant difference in VM scores between the two groups. Figures 8c–e illustrates the prototypical architecture of vasculogenic mimicry, in which short columnar tumor cells coalesce via intercellular adhesion (green fluorescence) to form tubular-like structures. The lumens lacked endothelial cells. Immunofluorescence staining of CD31 and E-cadherin in the two groups revealed that the samples with higher VM scores exhibited a correspondingly greater degree of vasculogenic mimicry within tumor tissues (Figure 8f). Overall, the data indicate that our VM score may be a potential biomarker for the degree of vasculogenic mimicry in hepatocellular carcinoma.

## 4 Discussion

Hepatocellular carcinoma (HCC) is the most common type of primary liver cancer (Vogel et al., 2022). Although surgical resection and liver transplant are recommended as the primary treatments for HCC, its overall survival remains poor (Lin et al., 2012). The advent of immunotherapy and targeted therapies represents a paradigm shift in cancer treatment (Girardi et al., 2020). Currently, there is substantial evidence that angiogenesis significantly contributes to the growth and spread of malignant tumors (Al-Ostoot et al., 2021). The activation of certain angiogenic pathways in HCC indicates their involvement in the progression and metastasis of the disease (Yao et al., 2023). An increasing number of anti-angiogenic drugs are being developed and applied to clinical treatments (Cao, 2016; Lopes-Coelho et al., 2021). Sorafenib is the first-line therapy for patients with HCC in an advanced stage (Feng et al., 2022). Moreover, bevacizumab, a monoclonal anti-vascular endothelial growth factor antibody, is regarded as the first-line drug in the treatment of advanced HCC worldwide (Kong et al., 2017). However, clinically, anti-angiogenic drugs cannot achieve satisfactory efficiency in some HCC patients (Wang et al., 2021). Maniotis et al. (1999) first put forward the concept of vasculogenic mimicry (VM) while studying human melanoma. VM is an epithelium-independent transportation system that can also deliver oxygen and nutrients to tumor cells. Recently, many researchers have suggested that this distinctive tumor microcirculation model was significantly correlated with poor overall survival and resistance to targeted drugs in digestive cancers (He et al., 2021; Haibe et al., 2020). Yang et al. (2016) reported that the presence of VM can predict poorer survival outcomes in cancer patients. Therefore, the identification of the relationship between VM and prognosis in HCC patients can help find novel prognostic factors and guide individualized treatment with targeted drugs.

Taking advantage of the TCGA database, we confirmed that previously reported VM-related genes were highly expressed in the tumor tissues of HCC patients. In addition, these genes were enriched in proteoglycans in the cancer pathway and the VEGF signaling pathway. These results reflected those of Wei X, who also found that the vessels formed in VM comprise endothelial tumor cells and are rich in outer matrix components (Fernandez-Cortes et al., 2019). Five hub genes, selected through LASSO Cox dimension reduction analysis, were deemed to be independent risk factors for the prognosis of HCC patients. As shown in Figure 2, KM curves demonstrated that these patients with such upregulated genes had much lower overall survival times. The results are consistent with those of Ahn S, who suggested that NOTCH1 expression might be an independent risk factor for overall survival in HCC patients (Ahn et al., 2013). This indicates that this molecule may be involved in the vasculogenic mimicry pathway. Our study of PPI network analysis also confirmed that MAPK1, MAPK3, NOTCH1, and TGFB1 are expressed at higher degrees during the vasculogenic mimicry process. Previous research, consistent with the findings of our study, has established that the hypoxic microenvironment can promote the process of VM by acting on the MAPK/ERK pathway through regulating the phenotype and function of cancer stem cells (Zhang et al., 2019). Subsequently, Benjakul N identified that NOTCH signaling regulates vasculogenic mimicry and promotes the epithelial-to-mesenchymal transition (Ren et al., 2022).

We calculated the VM score based on the risk coefficient of each hub gene. In previous research, VM was often found in aggressive tumors and was positively correlated with a poor prognosis (Ge and Luo, 2018; Hujanen et al., 2020; Liu et al., 2012). In addition, our results also demonstrated that patients with higher VM scores had a higher-grade TNM stage and poorer prognosis in the external database. In the GSE14520 dataset, KM curves and a clinical characteristic heatmap showed that patients in the high-risk group had a worse prognosis. By analyzing genes positively related to the VM score through GSEA, we found that these genes were enriched in the “NOTCH signaling pathway.” This finding is consistent with our previous study, which identified NOTCH1 as an independent prognostic predictor for HCC. Directly influencing vascular stability and vascular smooth muscle cell differentiation, NOTCH signaling is an essential component in the vascular development process (Kofler et al., 2011). It has been demonstrated that NOTCH1 signaling promotes HCC formation in a mouse model (Villanueva et al., 2012). Our study revealed that vasculogenic mimicry may be promoted in HCC through NOTCH signaling.

As shown in the figures from CIBERSORTx, there were significant differences in the distribution types and numbers of immune cells within HCC tumors. We speculated that the VM score might be related to immune infiltration in tumors. We further verified by comparing immune expression between the TCGA and GEO databases in the low- and high-risk groups. The results showed that the immune expression of regulatory T cells in the high VM score group was higher than that in the low VM score group in the GEO dataset. Previous studies have demonstrated that increased Tregs are correlated with disease progression through a variety of mechanisms (Fu et al., 2007). Our research found that the number of macrophages increased. Recent research has indicated that tumor-associated macrophages (TAMs), which infiltrate the majority of solid tumors, may facilitate tumor progression by promoting angiogenesis (Yang et al., 2020). M1 macrophages are a kind of macrophage that plays an inflammatory role. The tumor environment in the less-angiogenesis group is more likely to recruit M1 macrophages to suppress tumor progression and improve prognosis.

Moreover, it was validated in the GEO database that the infiltration of naïve CD4^+^ T cells was significantly linked to the expression of the VM score. Previously, we found that the number of regulatory T cells and macrophages increased in HCC tumors. This finding is consistent with that of Mo et al. (2022), who confirmed the connection between the macrophage-naïve CD4^+^ T-cell interaction and the Treg population. Macrophages induce naïve CD4^+^  T cells to differentiate into Tregs in cancer. Increasing infiltration of Tregs may promote tumor progression and lead to a poor prognosis. This provides an immunological basis for the use of the VM score to predict the prognosis of liver cancer patients.

An interesting finding was that the VM score was closely correlated with protein binding in molecular function analysis. According to a previous review, protein binding can enhance or detract from a drug’s performance in cancer treatment (Zeitlinger et al., 2011). The treatment of patients with HCC has been revolutionized by immune checkpoint inhibitors in recent years, and they have achieved satisfactory outcomes (Harkus et al., 2022). A recent study demonstrated that immune checkpoint inhibitors combined with anti-angiogenic drugs achieve better treatment efficiency than monotherapy (Song et al., 2020). However, choosing the appropriate immune checkpoint inhibitors remains a complex process. Additionally, we found that the VM score has the same expression pattern as inhibitory immune checkpoints such as CTLA4, HVEM, TIGIT, PD-1, CD47, and TIM-3 in the TCGA database. In both the TCGA and GEO datasets, HVEM, a tumor necrosis factor receptor, was positively correlated with the VM score. Among immune cells, HVEM is strongly expressed by Tregs (Tao et al., 2008). Upregulation of HVEM is closely associated with tumor progression and aggressiveness in many solid cancers. Yang et al. (2023) have demonstrated that VM-related genes could reflect the drug sensitivity of targeted drugs in lung adenocarcinoma. It is therefore likely that such connections exist between the VM score and drugs blocking the BTLA–HVEM interaction. An elevated VM score could be a reliable guide to applying these immune checkpoint inhibitors to HCC patients in their original treatment regimen. Hence, it could conceivably be hypothesized that there is a linkage between the VM score and the regulation activity of immune cells in the HCC tumor microenvironment. Our findings provide valuable information about potential targets and novel viewpoints for immunotherapy in HCC.

Our study, through *in vitro* validation, elucidated that the VM score can reflect the degree of vasculogenic mimicry of tumors. We applied RT-PCR to quantify the VM score; CD31/E-cadherin immunofluorescence staining revealed luminal structures composed solely of tumor cells. Through the utilization of endothelial cell-specific markers for labeling, we identified and precisely localized distinct categories of vessels within tumor tissues, delineating conventional vessels alongside vasculogenic mimicry. Our research showed a significant increase in VM structures in tumors with high VM scores. This indicates that VM scoring serves as an effective indicator for evaluating the vascular mimicry of tumors.

Vasculogenic mimicry has emerged as a critical mechanism of tumor vascularization in a variety of human cancers. In melanoma, VM contributes to tumor growth by forming blood vessel-like structures that circumvent the need for endothelial cells (Tan et al., 2022). In glioma, VM plays a pivotal role in tumor survival under hypoxic conditions, further enhancing its aggressive behavior (Maddison et al., 2023). In these cancers, VM is often correlated with adverse clinical outcomes, underscoring its potential as both a prognostic marker and a therapeutic target. Combining VM-targeted therapies with traditional anti-angiogenic treatments could offer a promising strategy for managing these aggressive tumors.

In addition to VM, other alternative mechanisms of tumor vascularization, such as vascular co-option and intussusceptive microvascular growth, have become increasingly recognized as key factors influencing tumor progression and metastasis. These processes may interact with or complement VM in sustaining the tumor’s vascular network. VM involves tumor cells mimicking endothelial cells to form blood vessel-like structures, providing a blood supply independent of endothelial cells. Vascular co-option occurs when tumors hijack pre-existing blood vessels from the host to support their growth, often observed in the early stages of tumor development (Kuczynski et al., 2019). Intussusceptive microvascular growth, in contrast, involves the splitting of existing blood vessels to form new microvascular structures, enhancing the complexity of the vascular network (Ribatti and Djonov, 2012). These mechanisms can operate simultaneously at different stages of tumor progression, with VM typically contributing to hypoxic regions of the tumor while vascular co-option predominates at the periphery. Moreover, the molecular regulation of these processes may overlap, with factors such as VEGF playing a role in modulating all three mechanisms (Lorenc et al., 2024). As a result, a combined therapeutic strategy targeting these alternative vascularization pathways may prove more effective than single-agent anti-angiogenic therapies, potentially inhibiting tumor growth and metastasis.

Furthermore, to explore the clinical application of the VM score, we constructed a nomogram based on the VM score and TNM stage to predict the 1-, 3-, and 5-year survival probabilities of patients. The AUCs of the time-dependent ROC curve showed that the nomogram achieved good performance. Calibration curves showed that the predicted probabilities of the nomogram closely aligned with the actual OS estimates in both the training and external validation groups. Of note, easily acquired factors make this predictive model more useful in the clinical setting.

## 5 Conclusion

In conclusion, we developed a prognostic signature based on MAPK1, MAPK3, VEGFA, NOTCH1, and TGFB1. The VM score and nomogram achieved satisfactory prognostic predictive performance in HCC patients. We confirmed that the VM score could be a reliable indicator for vasculogenic mimicry and a potential biomarker for immunotherapy.

## Data Availability

Publicly available datasets were analyzed in this study. These data can be found at: http://protal.gdc.cancer.gov and http://www.ncbi.nlm.nih.gov/geo(GSE14520).
