# Supplementary material for: A novel vasculogenic mimicry-related nomogram predicts prognosis in hepatocellular carcinoma
Source: Front Genet. 2025 Jul 7;16:1431624. doi: 10.3389/fgene.2025.1431624 (PMC12277141; doi:10.3389/fgene.2025.1431624)
Supplement: Supplementary file 1 [file Table1.docx]

| TableSI. Cox multivariate analysis | | | |  |
| --- | --- | --- | --- | --- |
| Characteristic | Hazard ratio | 95%CI | *P* |  |
| VM score | 2.330 | 1.407-3.858 | 0.001 |  |
|  |  |  |  |  |
| Gender | 1.014 | 0.687-1.496 | 0.944 |  |
|  |  |  |  |  |
| Age | 1.013 | 0.998-1.028 | 0.095 |  |
|  |  |  |  |  |
| TNM | 1.576 | 1.279-1.940 | < 0.001 |  |
|  |  |  |  |  |
