# Supplementary figures and images for: A novel vasculogenic mimicry-related nomogram predicts prognosis in hepatocellular carcinoma
Source: Front Genet. 2025 Jul 7;16:1431624. doi: 10.3389/fgene.2025.1431624 (PMC12277141; doi:10.3389/fgene.2025.1431624)

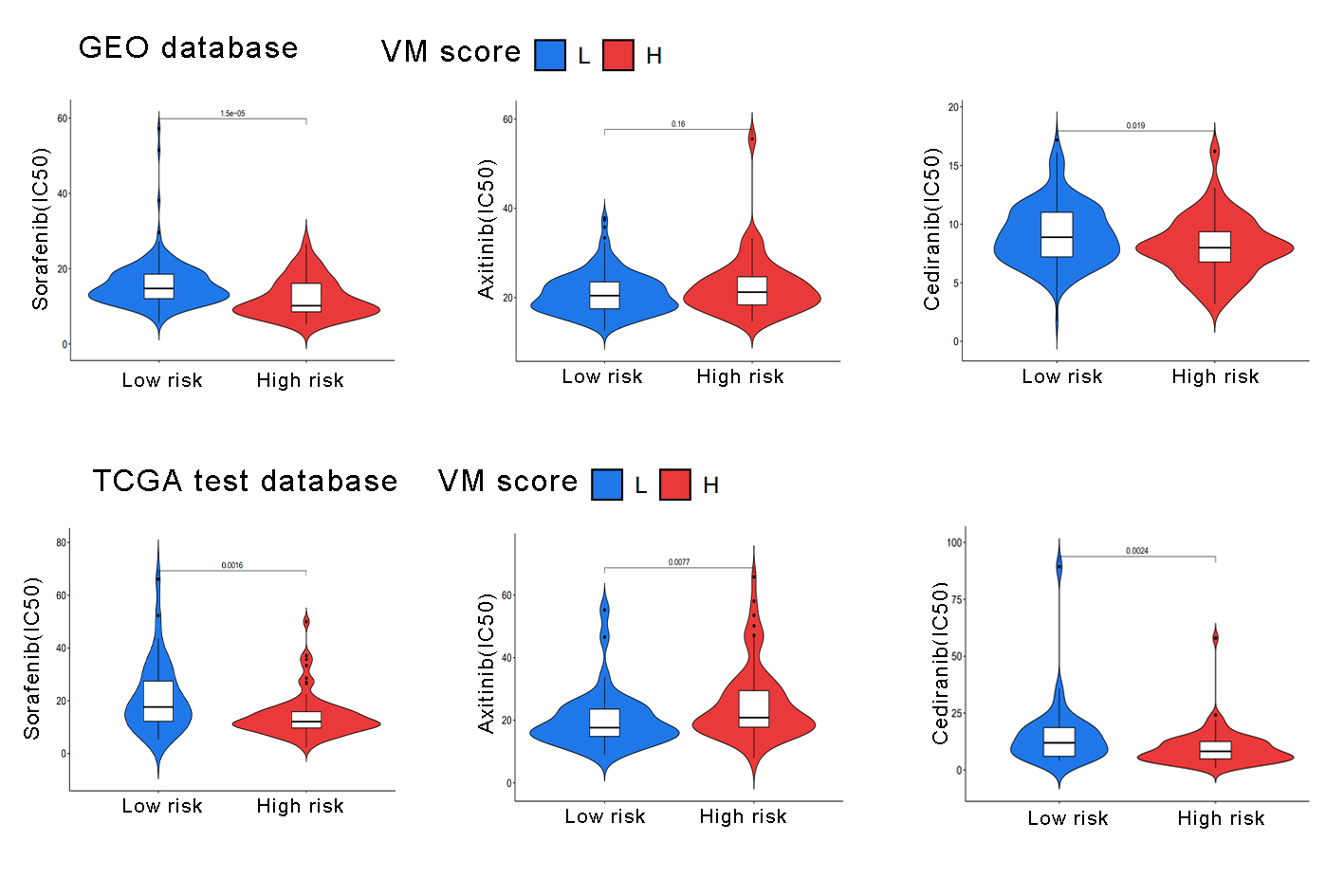

Supplement: Supplementary file 2 [file Image2.tif]

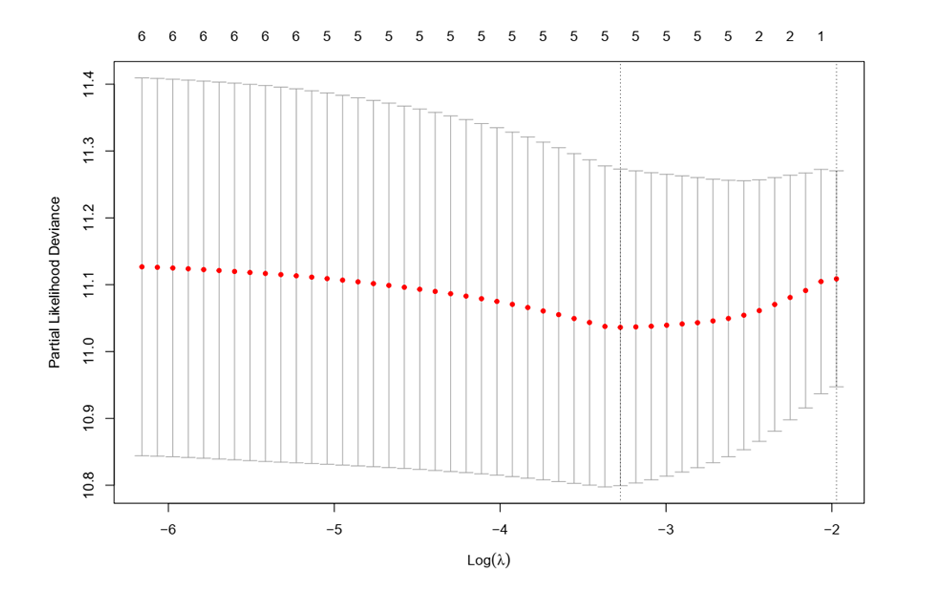

Supplement: Supplementary file 3 [file Image1.tif]
